# Impaired Itching Perception in Murine Models of Cholestasis Is Supported by Dysregulation of GPBAR1 Signaling

**DOI:** 10.1371/journal.pone.0129866

**Published:** 2015-07-15

**Authors:** Sabrina Cipriani, Barbara Renga, Claudio D’Amore, Michele Simonetti, Antonio Angelo De Tursi, Adriana Carino, Maria Chiara Monti, Valentina Sepe, Angela Zampella, Stefano Fiorucci

**Affiliations:** 1 Department of Medicine University of Perugia, Perugia, Italy; 2 Department of Surgery and Biomedical Sciences, University of Perugia, Perugia, Italy; 3 Department of Pharmacy, University of Salerno, Salerno, Italy; 4 Department of Pharmacy, University of Naples “Federico II”, Naples, Italy; Northeast Ohio Medical University, UNITED STATES

## Abstract

**Background & Aims:**

In cholestatic syndromes, body accumulation of bile acids is thought to cause itching. However, the mechanisms supporting this effect remain elusive. Recently, GPBAR1 (TGR5) a G-protein coupled receptor has been shown to mediate itching caused by intradermal administration of DCA and LCA. 6α-ethyl-3α, 7α-dihydroxy-24-*nor*-5β-cholan-23-ol (BAR502) is a non-bile acid dual ligand for FXR and GPBAR1.

**Methods:**

Cholestasis was induced in wild type and GPBAR1^-/-^ mice by administration of α-naphthyl-isothiocyanate (ANIT) or 17α-ethynylestradiol.

**Results.:**

In naïve mice skin application of DCA, TLCA, 6-ECDCA, oleanolic and betulinic acid induces a GPBAR1 dependent pruritogenic response that could be desensitized by re-challenging the mice with the same GPBAR1 agonist. In wild type and GPBAR1^-/-^ mice cholestasis induced by ANIT fails to induce spontaneous itching and abrogates scratching behavior caused by intradermal administration of DCA. In this model, co-treatment with BAR502 increases survival, attenuates serum alkaline phosphatase levels and robustly modulates the liver expression of canonical FXR target genes including OSTα, BSEP, SHP and MDR1, without inducing pruritus. Betulinic acid, a selective GPBAR1 ligand, failed to rescue wild type and GPBAR1^-/-^ mice from ANIT cholestasis but did not induced itching. In the 17α-ethynylestradiol model BAR502 attenuates cholestasis and reshapes bile acid pool without inducing itching.

**Conclusions:**

The itching response to intradermal injection of GPBAR1 agonists desensitizes rapidly and is deactivated in models of cholestasis, explain the lack of correlation between bile acids levels and itching severity in cholestatic syndromes. In models of non-obstructive cholestasis, BAR502 attenuates liver injury without causing itching.

## Introduction

Cholestasis is defined as the impairment of normal bile flow resulting either from a functional defect at the level of the hepatocyte or from obstruction at the bile duct level and might result from infections, drugs, and autoimmune, metabolic or genetic disorders [1–4]. The most common causes of cholestasis in adults are the primary biliary cirrhosis (PBC), the PBC-autoimmune hepatitis overlap syndrome, the primary sclerosing cholangitis (PSC) and the intrahepatic cholestasis of pregnancy (ICP) [1–4]. Under cholestatic conditions, a complex machinery of coordinated adaptive mechanisms is activated to counteract the potential hepatoxicity of bile components[5,6]. The nuclear receptor, farnesoid X receptor (FXR), a bile sensor mainly expressed in hepatocytes, is an essential component of the detoxification system that orchestrates liver protection against bile acid overload in condition of cholestasis[7–13]. Nevertheless, the role of FXR in cholestasis is controversial [8,12–15] since, FXR gene ablation or administration of an FXR antagonist[16] protects against liver damage caused by bile duct ligation (BDL), a severe model of obstructive choelstasis[14–15]. This protection is thought to be the result of alternative regulation of hepatic transporters, specifically MRP4, a basolateral transporter that is negatively regulated by FXR[9,13–16]. In addition, activation of FXR has been linked to induction/exacerbation of itching in cholestatic syndromes. Thus, in two phase II-III trials involving PBC patients administered a FXR ligand,.i.e. the semi-synthetic derivative of chenodeoxycholic acid (CDCA), the 6-ECDCA/obeticholic acid, itching was a common side effect being severe enough to cause treatment discontinuation in up to 40% of patients[17–20].

However, in addition to FXR, 6-ECDCA activates GPBAR1 (also known as TGR5) [21], a G-protein coupled receptor for secondary bile acids lithocolic acid (LCA) and deoxycholic (DCA) acid [22–26] and, experiments carried out in GPBAR1^-/-^ mice have identified this receptor as the putative mediator of itching caused by intradermal injection of LCA and DCA in mice [27].

**Fig 1:**
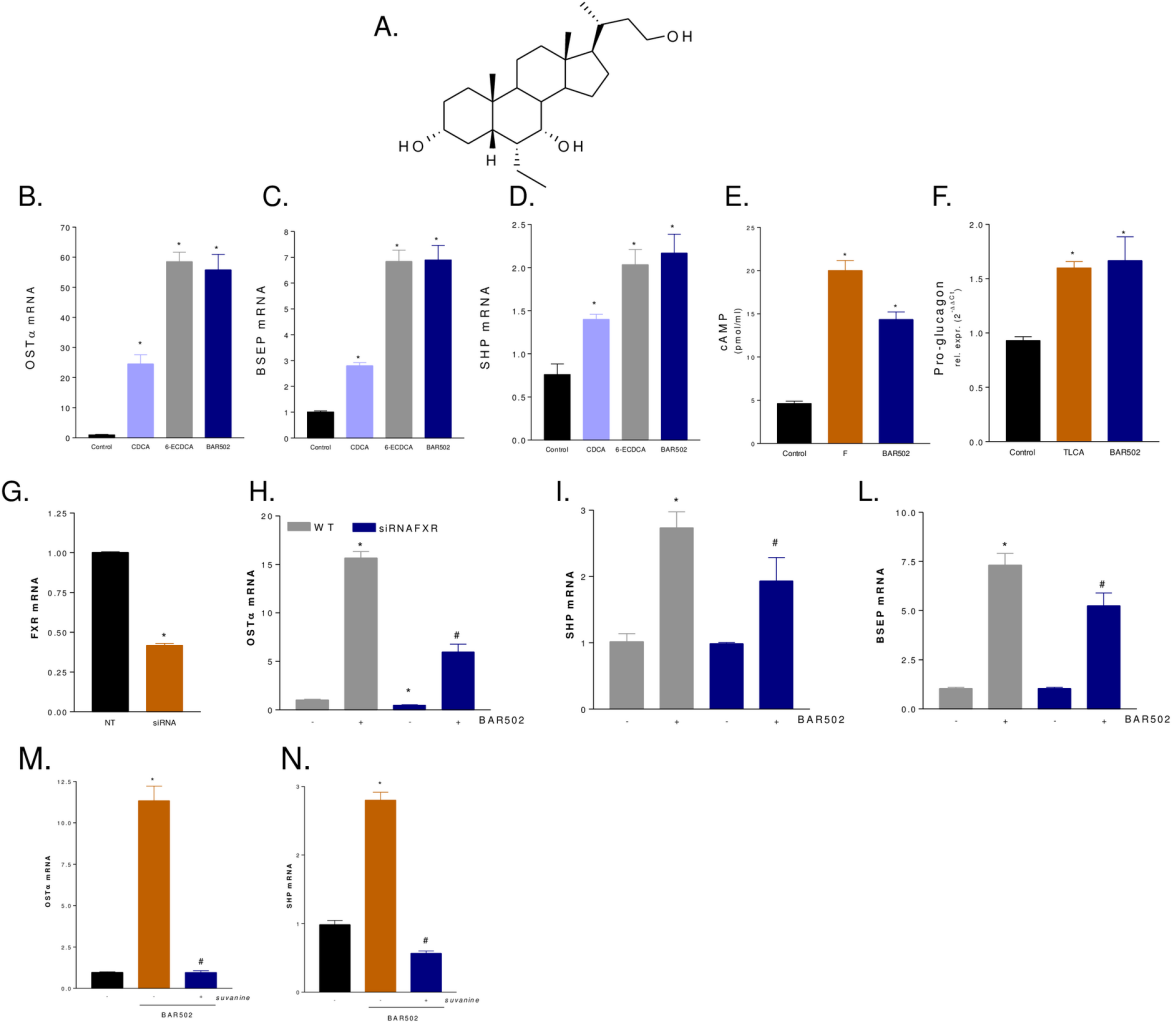
In vitro pharmacological characterization of BAR502. (A) Chemical structure of BAR502. (B-D) Serum starved HepG2 cells were stimulated 18 h with 10 M CDCA, 6-ECDCA or BAR501 and the relative mRNA expression of OSTα (B), BSEP (C) and SHP (D) was assayed by RT-PCR. Results are the mean ± SE of three experiments. *p<0.05 versus not treated cells (NT). (E) Serum starved THP-1 cells were stimulated with BAR501 or forskolin as indicated in materials and methods. At the end of stimulation intracellular cAMP levels were measured. (F) Glutag cells were stimulated 18 h with 10 M TLCA or BAR501 and the relative mRNA expression of pro-glucagon was assayed by RT-PCR. Results are the mean ± SE of three experiments. *p<0.05 versus not treated cells (NT). (G-L) Effect of FXR silencing on activity of BAR502 on FXR target genes. HepG2 cells were left untransfected or transfected with a cocktail of four siRNA directed against FXR. 48 hours post transfection cells were stimulated 18 with BAR502 (10 M) and the relative mRNA expression of FXR (G), OSTα (H), SHP (I) and BSEP (L) were assayed by RT-PCR. (M-N) Effect of FXR antagonism by suvanine. Serum starved HepG2 cells were stimulated 18 hours with BAR502 (10 M) or with the combination of BAR 502 plus suvanine (50 M). At the end of stimulation the relative mRNA expression of OSTα (M) and SHP (N) were assayed by RT-PCR.

The 6α-ethyl-3α, 7α-dihydroxy-24-*nor*-5β-cholan-23-ol (NorECDCOH christened BAR502) is a non bile acid steroidal dual ligand for FXR and GPBAR1 (Fig 1A) [28]. Here, we report the pharmacological characterization of this agent in rodent models of cholestasis.

By using GPBAR1^-/-^ mice we demonstrate that while this receptor mediates the acute pruritogenic effects of intradermal injection of bile acids and other natural GPBAR1 ligands in naïve mice, this pathway appears deactivated in rodent models of cholestasis highlighting the complex interplay between bile acids and their receptors in the determinism of itching and providing an additional explanation for the lack of correlation between plasma bile acids and itching in clinical setting.

## Materials and Methods

### Chemistry of BAR502

Design and synthesis of BAR502 (6α-ethyl-3α, 7α -dihydroxy-24-*nor*-5β-cholan-23-ol) has been described previously[28].

### Transactivation assay

Activity of BAR502 toward GPBAR1 and FXR was assessed *in vitro* by transactivation assays, measurement of intracellular concentrations of cAMP and expression of GPBAR1 and FXR target genes in cells was described elsewhere[28]. HepG2, THP1 and HEK293T cells were from ATCC. HepG2 cells were cultured in E-MEM supplemented with 10% FBS, 1% glutamine, 1% penicillin/streptomycin. GLUTAg cells, a murine intestinal endocrine cell type, were kindly donated by Dr. D. J. Drucker, Banting and Best Diabetes Centre, University of Toronto, Toronto, Canada. GLUTAg cells were cultured in D-MEM, supplemented with 10% FBS, 1% glutamine, and 1% penicillin/streptomycin. THP-1 cells were cultured in RPM-I supplemented with 10% FBS, 1% glutamine, and 1% penicillin/streptomycin. Affinity of BAR502 versus FXR and GPBAR1 was calculated by performing dose response curves in transactivation experiments. For FXR mediated transactivation, HepG2 cells were plated at 5 × 104 cells/well in a 24 well plate. Cells were transfected with 200 ng of the reporter vector p(hsp27)-TK-LUC containing a FXR response element (IR1) cloned from the promoter of heat shock protein 27 (hsp27), 100 ng of pSG5-FXR, 100 ng of pSG5-RXR, and 100 of pGL4.70 (Promega), a vector encoding the human Renilla gene. For GPBAR1 mediated transactivation, HEK-293T cells were plated at 1 × 10^4^ cells/well in a 24 well-plate and transfected with 200 ng of pGL4.29 (Promega), a reporter vector containing a cAMP response element (CRE) that drives the transcription of the luciferase reporter gene luc2P, with 100 ng of pCMVSPORT6-human GPBAR1, and with 100 ng of pGL4.70. At 24 h post-transfection, HepG2 and HEK293T cells were incubated with increasing concentrations of BAR502 (0.1, 0.5, 1, 10 and 50 μM) for 18 hours and luciferase activities were assayed and normalized against the Renilla activities. Efficacy of BAR502 against FXR in HepG2 cells was calculated by comparing the magnitude of transactivation elicited by BAR502 (10 μM) with that of 6-ECDCA (10 μM), which was set to 100%. Efficacy of BAR502 against GPBAR1 in HEK293T cells was calculated by comparing the maximal response elicited by BAR502 (10 μM) with that obtained with TLCA (10 μM), which was set to 100%.

### Cellular modulation of FXR expression/activity with small interfering RNA

HepG2 cells were transfected with a set of 4 siRNA cloned into pGFP-V-RS plasmids (cat. TG320627 – Origene Technologies) using Fugene HD transfection reagent (Promega) as described by the manufacturer. Forty-eight hours post transfection cells were stimulated with 10 μM BAR502 and incubated for 18 h at 37°C at 5% CO_2_. At the end of experiment the relative mRNA expression of OSTα, BSEP and SHP was assayed by Real-Time PCR.

### Pharmacological inhibition of FXR activity by suvanine

Serum starved HepG2 cells were incubated 18 hours with 10 μM BAR502 alone or in combination with 50 μM suvanine, a previously described marine sponge sesterterpene endowed with a FXR antagonistic activity. At the end of experiment the relative mRNa expression of OSTα and SHP was assayed by Real-Time PCR[29].

### Assay of cAMP concentrations in THP-1 cells

cAMP generation in THP-1 cells was assayed using the Direct Cyclic AMP enzyme immuno-assay kit (Arbor Assay cat. no. K019-H1). THP-1 cells were serum starved overnight and then stimulated for 30 min with 10 μM forskolin (F) or BAR502.

### Animals and protocols

GPBAR1 null mice (GPBAR1-B6 = GPBAR12/2 mice, generated directly into C57BL/6NCrl background), and congenic littermates on C57BL/6NCrl mice were kindly gifted by Dr. Galya Vassileva (Schering-Plough Research Institute, Kenilworth). Mice were housed under controlled temperatures (22°C) and photoperiods (12:12-hour light/dark cycle), allowed unrestricted access to standard mouse chow and tap water and allowed to acclimate to these conditions for at least 5 days before inclusion in an experiment. The study was conducted in agreement with the Italian law and the protocol was approved by a ethical committee of University of Perugia and by a national committee of Ministry of Health (N°245/2013-B).

### Scratching test

GPBAR1^-/-^ mice and their congenic littermates (male, 8–12 weeks of age) were used for this studies. The fur at the base of the neck was shaved, and mice were placed in individual cylinders on a glass shelf. A circumference of approx. 0.5 cm of diameter was drawn in the neck and test agents injected in this area. Mice were acclimatized to the experimental room, restraint apparatus and investigators for 2-hour periods on 2 successive days before experiments. Scratching behavior was quantified by 2 observers unaware of tested agents or genotypes. A scratch was defined as lifting the hind limb to the injection site and then a placing of the paw on the floor, regardless of the number of strokes. If counts differed by greater than 5 scratches over a 30-minute period, both observers reevaluated the records. Results were expressed as the number of scratching events during 30 or 60 minutes of observation. Tested agents DCA (25 μg), TLCA (25 μg), UDCA (25 μg), and BAR502 (25 μg), or with betulinic acid (50 μg), oleanolic acid (50 μg) and PAR-2 agonist peptide SLIGRL (1–6) amide (50 μg) [24–27]were administered by intradermal injection at the nape of the neck. LCA and DCA were dissolved in DMSO and the other agents in 0.9% NaCl (10 μl).

In another experimental setting GPBAR1^-/-^ mice and their congenic littermates were treated with α-naphtylisothiocyanate (ANIT), 25 mg/kg, *per os*, dissolved in olive oil or olive oil alone (control mice) or with the combination of ANIT plus BAR502 (15 mg/Kg once a day, *per os*) for 10 days. At day 5 spontaneous scratching was evaluated for 60 minutes as well as after subcutaneous injection of 25 μg DCA. In another experimental setting GPBAR1^-/-^ mice and their congenic littermates were treated 5 days with ANIT (Sigma-Aldrich) dissolved in olive oil (25 mg/kg, *per os*) or olive oil alone (control mice) or with the combination of ANIT plus betulinic acid (15 mg/Kg once a day, *per os*). At day 5 spontaneous scratching was evaluated for 60 minutes. Control mice were the same in both experimental sets. Serum levels of total bilirubin, aspartate aminotransferase (AST) and alkaline phosphatase were measured by routine clinical chemistry testing performed on a Hitachi 717 automatic analyzer. For the estrogen model, wild type C57BL6 mice were administered 10 mg/Kg i.p. with 17α-Ethynylestradiol (17αE_2_) (cat. E4876-1G Sigma–Aldrich) dissolved in PEG or PEG alone (control mice) or the combination of 17αE_2_ and BAR502 (15 mg/Kg daily, *per os*) for 8 days[30]. At the end of the study the spontaneous scratching and scratching induced by s.c. injection of 25 μg DCA was recorded. Gallbladder weight and serum levels of bilirubin and alkaline phosphatase were also measured.

Throughout the studies animals were visually assessed at least twice a day from Monday to Friday and once a day over the week end by investigators (S.C., M.S. and A.A.DeT) and by highly trained animal facility personnel’s including animal facility’s veterinarian. Animals were weighted daily and sacrificed at indicated time points or when their clinical conditions become critical as assessed by a reduction of body weight higher than 25% of basal body weight in 7 days. In addition, animals were sacrificed when at the daily evaluation they demonstrate inability to rise or ambulate. Mice were euthanized by an overdose of sodium pentobarbital (>100 mg/kg i.p.).

### Liver histopathology

For histological examination, portions of the right and left liver lobes from each animal were fixed in 10% formalin, embedded in paraffin, sectioned, and stained with hematoxylin and eosin.

### RNA extraction and Real-Time PCR

Total RNA was isolated using the TRIzol reagent according to the manufacturer’s specifications (Invitrogen). One microgram of purified RNA was treated with DNaseI for 15 min at room temperature, followed by incubation at 95°C for 3 min in the presence of 2.5 mmol/L EDTA. The RNA was reverse transcribed with Superscript III (Invitrogen) in 20 μL reaction volume using random primers. For Real Time PCR, a 10 ng template was dissolved in 25 μL containing 200 nmol/L of each primer and 12.5 μL of 2×SYBR FAST Universal ready mix (Invitrogen). All reactions were performed in triplicate, and the thermal cycling conditions were as follows: 2 min at 95°C, followed by 40 cycles of 95°C for 10 s and 60°C for 45 s in iCycler iQ instrument (Biorad). The relative mRNA expression was calculated and expressed as 2−(ΔΔCt). Forward and reverse primer sequences were the following: human GAPDH, gaaggtgaaggtcggagt and catgggtggaatcatattggaa; human OSTα, tgttgggccctttccaatac and ggctcccatgttctgctcac; human BSEP, gggccattgtacgagatcctaa and tgcaccgtcttttcactttctg; human SHP, gctgtctggagtccttctgg and ccaatgatagggcgaaagaagag; mouse GAPDH, ctgagtatgtcgtggagtctac and gttggtggtgcaggatgcattg; mouse Pro-glucagon, tgaagacaaacgccactcac and caatgttgttccggttcctc; mGAPDH: gaaggtgaaggtcggagt and catgggtggaatcatattggaa; mFXR: tacatgcgaagaaagtgtcaaga and actgtcttcattcacggtctgat; mSHP: gctgtctggagtccttctgg and ccaatgatagggcgaaagaagag; mOSTα: tgttgggccctttccaatac and ggctcccatgttctgctcac; mBSEP: gggccattgtacgagatcctaa and tgcaccgtcttttcactttctg; mCyp7α1: caccttgaggacggttccta and cgatccaaagggcatgtagt; mMDR1: gtggggcaagtcagttcatt and tcttcacctccaggctcagt; mNTCP: tcaagactcccaaggataaaaca and atgtggaaatgctggagaaa; mIL1β: ggacaagctgaggaagatgc and tcgttatcccatgtgtcgaa; mIL6: aggagacttgcctggtgaaa and caggggtggttattgcatct; mTNFα: aacctcctctctgccatcaa and ggaagacccctcccagatag.

### Bile acids determination, liquid chromatography and mass spectrometry

Bile acids determination, liquid chromatography and mass spectrometry were performed as previously described[31].

### Statistical Analysis

All values are mean ± Standard Error (SE) of number (n) observations per group. Comparisons of more than two groups were made with a one-way ANOVA with post-hoc Tukey’s test. Comparison of two groups was obtained by the Student’s t-test for unpaired data when appropriate.

## Results

### BAR502 is a dual FXR and GPBAR1 ligand

Exposure of HepG2 cells overexpressing FXRE to BAR502 caused a concentration-dependent transactivation of FXRE with an EC_50_ of ≈ 2 μM, while the efficacy of BAR502 in FXRE transactivation at the concentration of 10 μM was 335% compared to that of 6-ECDCA (Table 1). In HEK293 cells cotransfected with a CRE responsive element (Table 1), BAR502 caused a concentration dependent transactivation of CRE with an EC_50_ of ≈ 400 nM, while the efficacy at 10 μM was 79.5% compared to TLCA (n = 4), making this agent a potent FXR agonist. To further investigate whether BAR502 elicits FXR and GPBAR1 agonistic activities in cells not overexpressing the two receptors, HepG2 and GLUTAg cells were incubated with BAR502, CDCA, 6-ECDCA or TLCA and expression of target genes assessed by RT-PCR. In HepG2 cells, BAR502 (10 μM) induced the expression of OSTα, BSEP and SHP, three FXR target genes. The magnitude of the effect of BAR502 was similar to that of 6-ECDCA (10 μM) (Fig 1B–1D; n = 3 in triplicate; P<0.05 versus control cells).

**Table 1:** BAR502 is dual FXR and GPBAR1 ligand.

GPBAR1*		FXR**	
Affinity(μM)	Efficacy (% vs TLCA)	Affinity (μM)	Efficacy (% vs 6-ECDCA)
0.4 ± 0.1	79.5 ± 11.8	2.0 ± 0.2	325.0 ± 38.1

Data are mean ± SE of 3 experiments in duplicate

* Activity of BAR502 toward GPBAR1 in a reporter assay was assessed in HEK293T cells transfected with a cAMP responsive element (CRE) cloned upstream to the luciferase gene (see Materials and Methods). For calculation of efficacy data, maximal transactivation of CRE caused by BAR502 (10 μM) was compared to maximal transactivation caused by TLCA (10 μM).

** Activity of BAR502 toward FXR in a reporter assay was assessed in HepG2 cells transfected with a FXR responsive element (FRE) cloned upstream to the luciferase gene (see Materials and Methods). For calculation of efficacy data, maximal transactivation of FRE caused by BAR502 (10 μM) was compared to maximal transactivation caused by 6-ECDCA (10 μM).

The GLUTAg cells are a murine cell line that show similarities with human intestinal L cells: these cells respond to GPBAR1 activation by accumulating cAMP and releasing GLP-1[28]. In this system, BAR502 (10 μM) effectively stimulates cAMP accumulation and the effect was comparable with that of forskolin (Fig 1E, n = 3 in triplicate; p<0.01 versus control). In addition, BAR502 caused GLP-1 release with the same potency of TLCA (Fig 1F, n = 3 in triplicate; p<0.01 versus control).

The treatment of HepG2 cells with an anti-FXR siRNA (Fig 1G, n = 3 in triplicate; *p<0.01 versus not transfected cells) resulted in a partial reduction of FXR gene, as well as reduced expression of OSTα, SHP and BSEP mRNAs induced by exposure to BAR502 (Fig 1H–1L, n = 3 in triplicate; *p<0.01 versus not transfected cells; #p<0.01 versus BAR501 stimulated cells). Similarly co-administration of HepG2 cells with both BAR502 and suvanine, a previously described FXR antagonist,^29^ reversed the induction of FXR target genes OSTα and SHP mediated by BAR502 (Fig 1M and 1N, n = 3 in triplicate; *p<0.01 versus not treated cells; #p<0.01 versus BAR501 stimulated cells).

Taken together these data demonstrate that BAR502 is a preferential FXR agonist in a transactivation assay and effectively modulates FXR and GPBAR1 target genes.

### Itching induced by intradermal injection of secondary bile acids is GPBAR1 dependent

**Fig 2:**
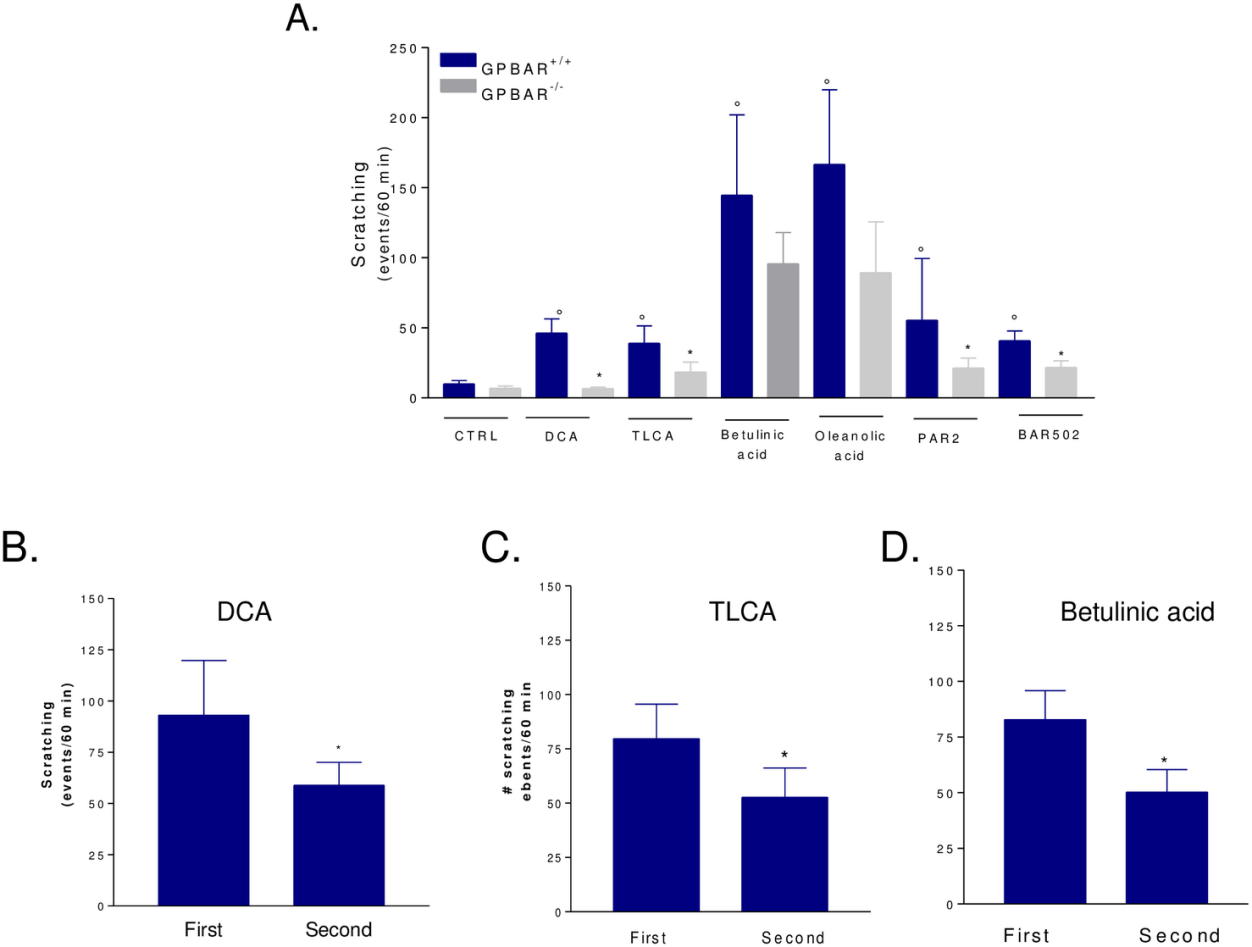
Effects of BAR502 on scratching behavior in GPBAR1^+/+^ and GPBAR1^-/-^ mice. (A) GPBAR1^**+/+**^ and GPBAR1^**-/-**^ mice were subjected to intradermal injection of DCA, TLCA, betulinic acid, oleanolic acid, PAR-2 agonist and BAR502. Each agent was tested at the dose of 25 μg. Results are expressed as the number of scratching events during 60 minutes of observation. Results are the mean ± SE of 4–8 mice per group. °p<0.05 versus control group (CTRL); *p<0.05 versus GPBAR1^**+/+**^ mice. B-D. Desensitization of the itching pathway by a second administration of a GPBAR1 ligand. Animals were administered DCA, TLCA or betulinic acid at the dose of 50 μg. After counting scratching for 60 minutes animals were rechallenged with a second dose of the given agonist and the scratching response was recorded for a further 60 minutes of observation. Data are mean ± SE of 6–7 animals per group.] p>0.01 versus first dose.

Since in naïve mice secondary bile acids cause itching through the activation of GPBAR1[27], we have investigated whether BAR502 induces an itching. For this purpose GPBAR1^+/+^ and GPBAR1^-/-^ mice were injected intradermally with BAR502 and compared its activity with DCA and TLCA, betulinic and oleanolic acid [27]. As summarized in Fig 2A, itching caused by TLCA and DCA, betulinic acid and oleanolic acid was robustly attenuated by GPBAR1 gene ablation (*p<0.05). To further examine the selectivity of these interactions, we have administered wild type and GPBAR1^-/-^ mice with SLIGRL-NH_2_, a PAR2 agonist. Of relevance this agent induced pruritus in both GPBAR1^+/+^ and GPBAR1^-/-^ mice indicating that its pruritogenic activity is GPBAR1 independent [27]. As illustrated in Fig 2B, intradermal injection of BAR502 induced a GPBAR1 dependent itching. Similarly to BAR502, 6-ECDCA caused itching that was GPBAR1 dependent (S1 Fig, n = 4; p<0.01 versus naïve mice or GPBAR1 wild type mice).

Because transduction of pruritogenic signals behind GPBAR1 is mediated by a number of signaling mechanisms and receptors located at various levels in the skin, spinal cord and brain [27], we have then examined whether the pruritogenic response to GPBAR1 agonism was attenuated by a second challenge with the same agent. The results of these experiments, shown in Fig 2C–2E, demonstrate that the pruritogenic response induced by DCA, TLCA and betulinc acid is reduced by approx. 50% when animals are rapidly challenged with a second doses of the same agonist (P<0.01; n = 6–7 animals per group). In contrast, the itching response to oleanolic acid could not be desensitized (not shown) likely due to the activation of additional receptors.

### GPBAR1 gene ablation worsens the liver injury induced by ANIT

**Fig 3:**
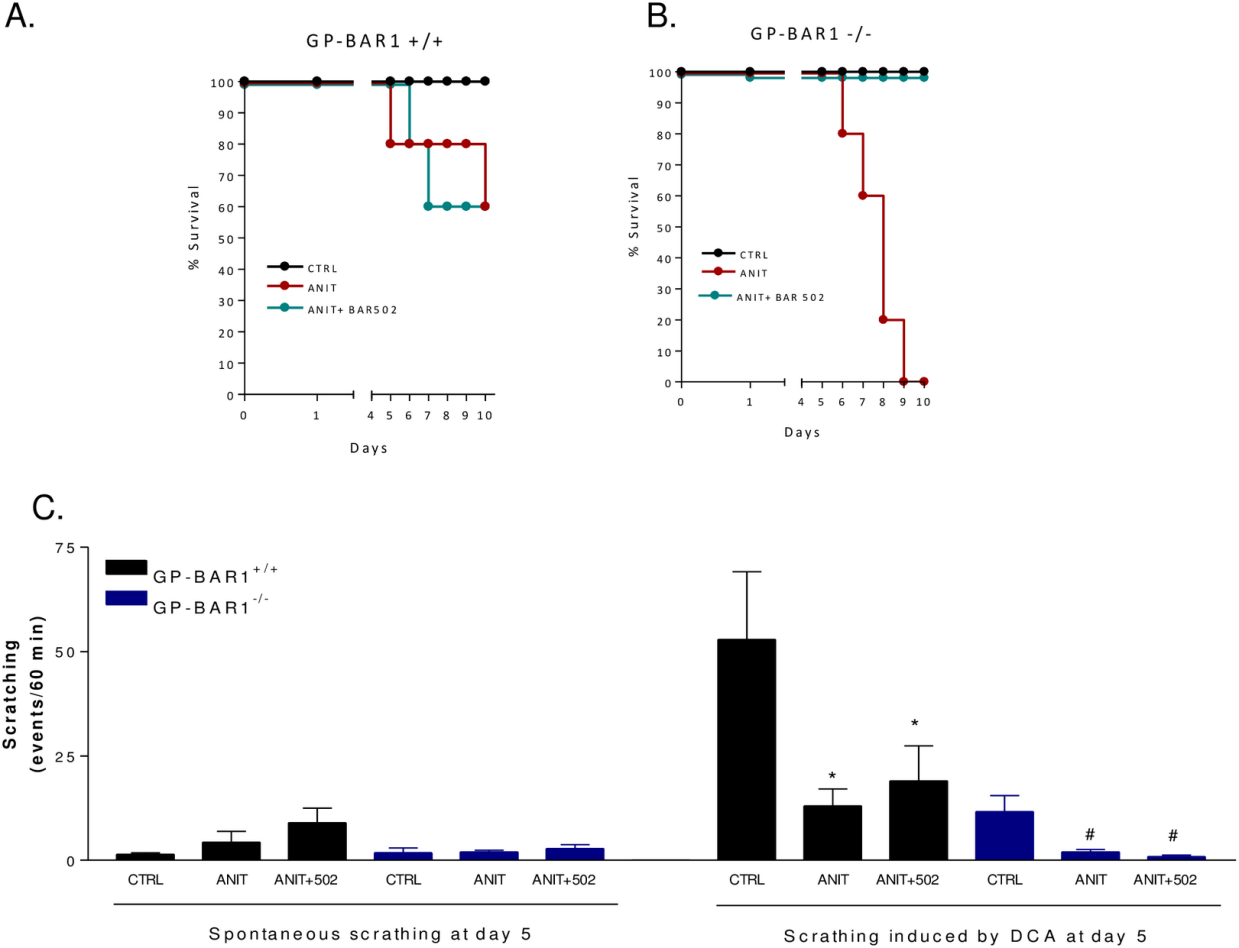
Effects of BAR502 on scratching behavior in GPBAR1^+/+^ and-^/-^ mice administered with ANIT. GPBAR1^**+/+**^ and-^**/-**^ mice were treated for 10 days with ANIT or with the combination of ANIT plus BAR502. At the end of the treatments mice were subjected to intradermal injection of DCA. (A-B) Survival of GPBAR1^**+/+**^ and-^**/-**^ mice in response to ANIT administration. (C) Effect of ANIT on spontaneous scratching and scratching induced by DCA. Scratching tests were performed at day 5. Results are expressed as the number of scratching events during 60 minutes of observation. Results are the mean ± SE of 4–8 mice per group. *p<0.05 versus GPBAR1^**+/+**^ mice subjected to intradermal injection of DCA. #p<0.05 versus GPBAR1^**-/-**^ mice subjected to intradermal injection of DCA.

Because the relation of itching and GPBAR1 expression in cholestasis is unknown, we have next investigated if BAR502 trigger itching in a rodent model of cholestasis [31]. For this purpose, wild type and GPBAR1^-/-^ mice were administered with ANIT, 25 mg/kg/day, alone or in combination with BAR502, 15 mg/kg/day, for 10 days. Exposure to ANIT caused a severe illness resulting in 100% lethality at day 10 in GPBAR1^-/-^ mice. Administration of BAR502 rescued GPBAR1^-/-^ mice from death (Fig 3A and 3B, p<0.05 versus ANIT alone). A scratching test was performed on day 5. As shown in Fig 3C, at this time point both GPBAR1^+/+^ and GPBAR1^-/-^ mice exhibited a low frequency of spontaneous scratching and BAR502 failed to trigger itching. Furthermore, exposure to ANIT blunted the number of scratching bouts caused by the intradermal administration of DCA in both GPBAR1^+/+^ and GPBAR1^-/-^ mice treated with ANIT alone or ANIT in combination with BAR502 (Fig 3C; n = 6–8 mice; P<0.05 versus basal).

**Fig 4:**
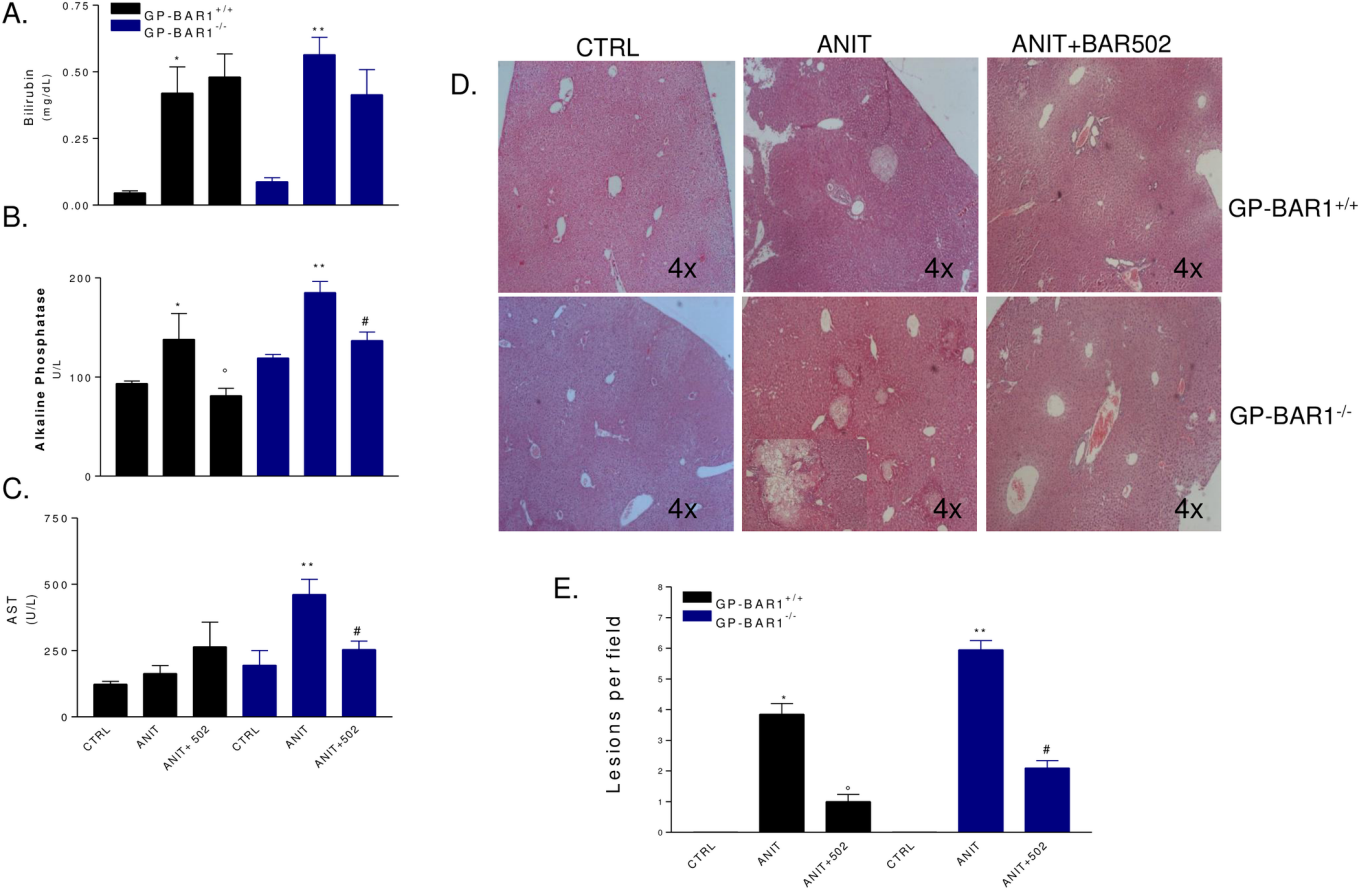
Dual FXR/TGR5 agonist BAR502 improves liver injury in both GPBAR1^+/+^ and GPBAR1^-/-^ mice. GPBAR1^**+/+**^ and GPBAR1^**-/-**^ mice were treated for 10 days with ANIT or with the combination of ANIT plus BAR502. Serum levels of (A) bilirubin, (B) Alkaline phosphatase and (C) AST. Results are the mean ± SE of 4–8 mice per group. *p<0.05 versus GPBAR^**+/+**^ mice. °p<0.05 versus GPBAR1^**+/+**^ mice treated with ANIT. **p<0.05 versus GPBAR1^**-/-**^ mice. #p<0.05 versus GP-BAR^**-/-**^ mice administered with ANIT. (D) Representative histological pictures of H&E-stained livers. (E) Analysis of the number of lesions per field. Results are the mean ± SE of 4–8 mice per group. *p<0.05 versus GPBAR1^**+/+**^ mice. °p<0.05 versus GPBAR1^**+/+**^ mice treated with ANIT. **p<0.05 versus GPBAR1^**-/-**^ mice. #p<0.05 versus GPBAR1^**-/-**^ mice administered with ANIT.

**Fig 5:**
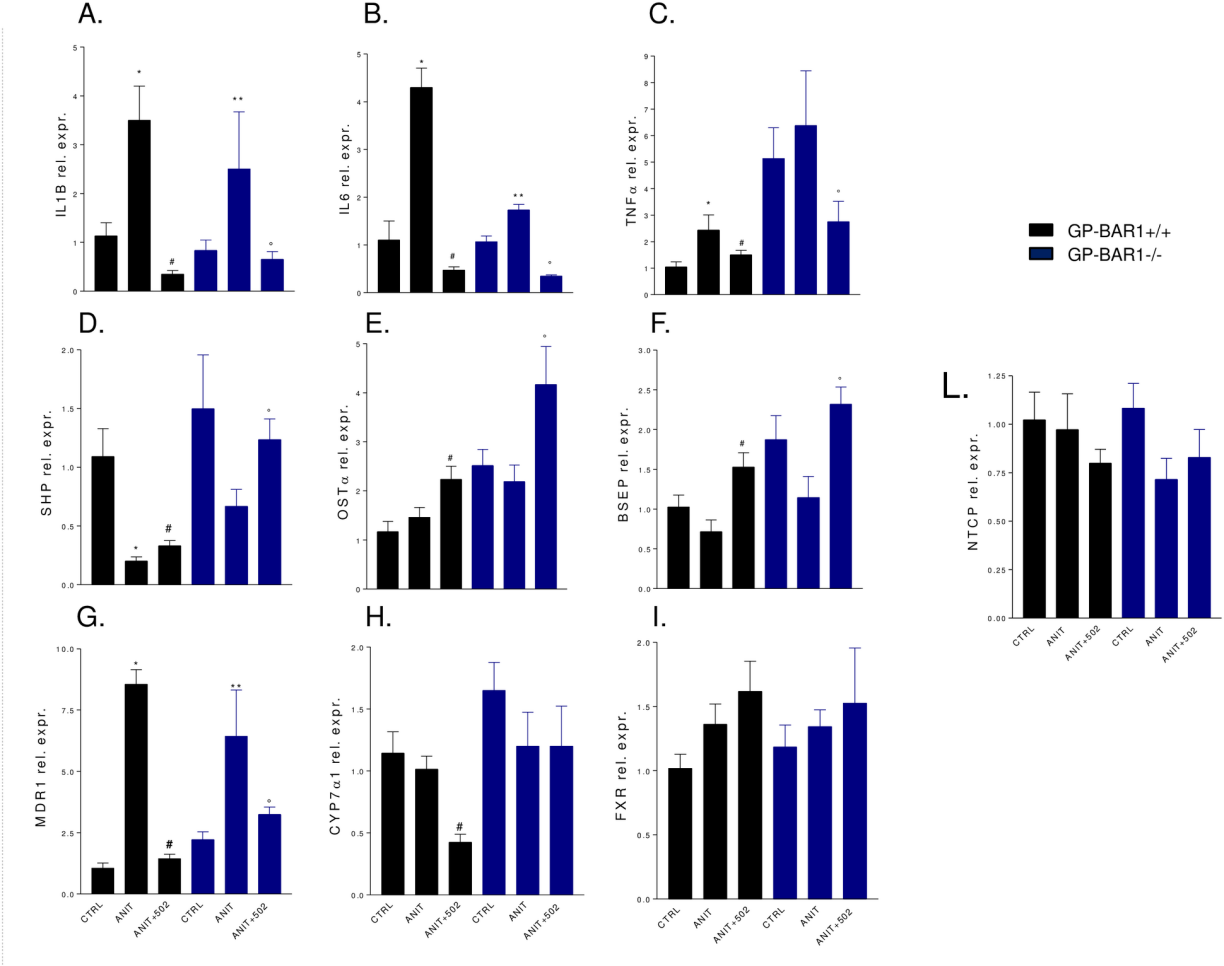
BAR502 reduces inflammatory cytokines and regulates canonical FXR target genes during ANIT induced cholestasis. GPBAR1^**+/+**^ and GPBAR1^**-/-**^ mice were treated for 10 days with ANIT or with the combination of ANIT plus BAR502. Quantitative Real-Time PCR of (A) IL1β, (B) IL6, (C) TNFα, (D) SHP, (E) OSTα, (F) BSEP, (G) MDR1, (H) Cyp7α1, (I) FXR and (L) NTCP. Results are the mean ± SE of 4–8 mice per group. *p<0.05 versus GPBAR1^**+/+**^ mice. #p<0.05 versus GPBAR1^**+/+**^ mice treated with ANIT. **p<0.05 versus GPBAR1^**-/-**^ mice. °p<0.05 versus GPBAR1^**-/-**^ mice administered ANIT.

Because the high mortality caused by ANIT in GPBAR1^-/-^ mice a second set of experiment was performed to obtain biochemical data and GPBAR1^-/-^ mice were sacrificed at day 5. As shown in Fig 4A–4C, administering wild type mice with ANIT increases plasma levels of bilirubin and alkaline phosphatase but not AST (n = 6–8 mice; p<0.05). GPBAR1 gene ablation worsened the severity of liver disease caused by ANIT, as assessed by measuring bilirubin, alkaline phosphatase and AST levels (Fig 4A–4C, p<0.05). BAR502 attenuated changes in alkaline phosphatase and reduced AST levels in GPBAR1^-/-^ animals while it had no effect on bilirubin plasma levels (Fig 4A–4C; p<0.05). Histopathology examination demonstrates severe liver necrosis in GPBAR1^-/-^ mice exposed to ANIT (Fig 4D). This pattern was attenuated by treating GPBAR1^+/+^ and GPBAR1^-/-^ mice with BAR502 (Fig 4D and 4E, p<0.05). As shown in Fig 5, exposure to ANIT increased the liver expression of IL-1β, IL-6 and TNFα in both GPBAR1^+/+^ and GPBAR1^-/-^ mice (Fig 4A–4C; p<0.05). This pattern was reversed by administering mice with BAR502 (Fig 5A–5C; p<0.05), thus indicating that the anti-inflammatory effect of this agent is GPBAR1 independent. Confirming this view BAR502 behaves as a FXR agonist in vivo, increasing the liver expression of SHP, OSTα and BSEP, three FXR canonical target genes, in both GPBAR1^+/+^ and GPBAR1^-/-^ mice (Fig 5D–5F; p<0.05). Similarly, BAR502 attenuated changes in MDR1 expression in both wild type and GPBAR1 null mice (Fig 5G, p<0.05) but had no effect on Cyp71 mRNA expression in GPBAR1 null mice (Fig 5H).

### Betulinic acid, a GPBAR1 ligand, fails to rescue from ANIT induced injury

**Fig 6:**
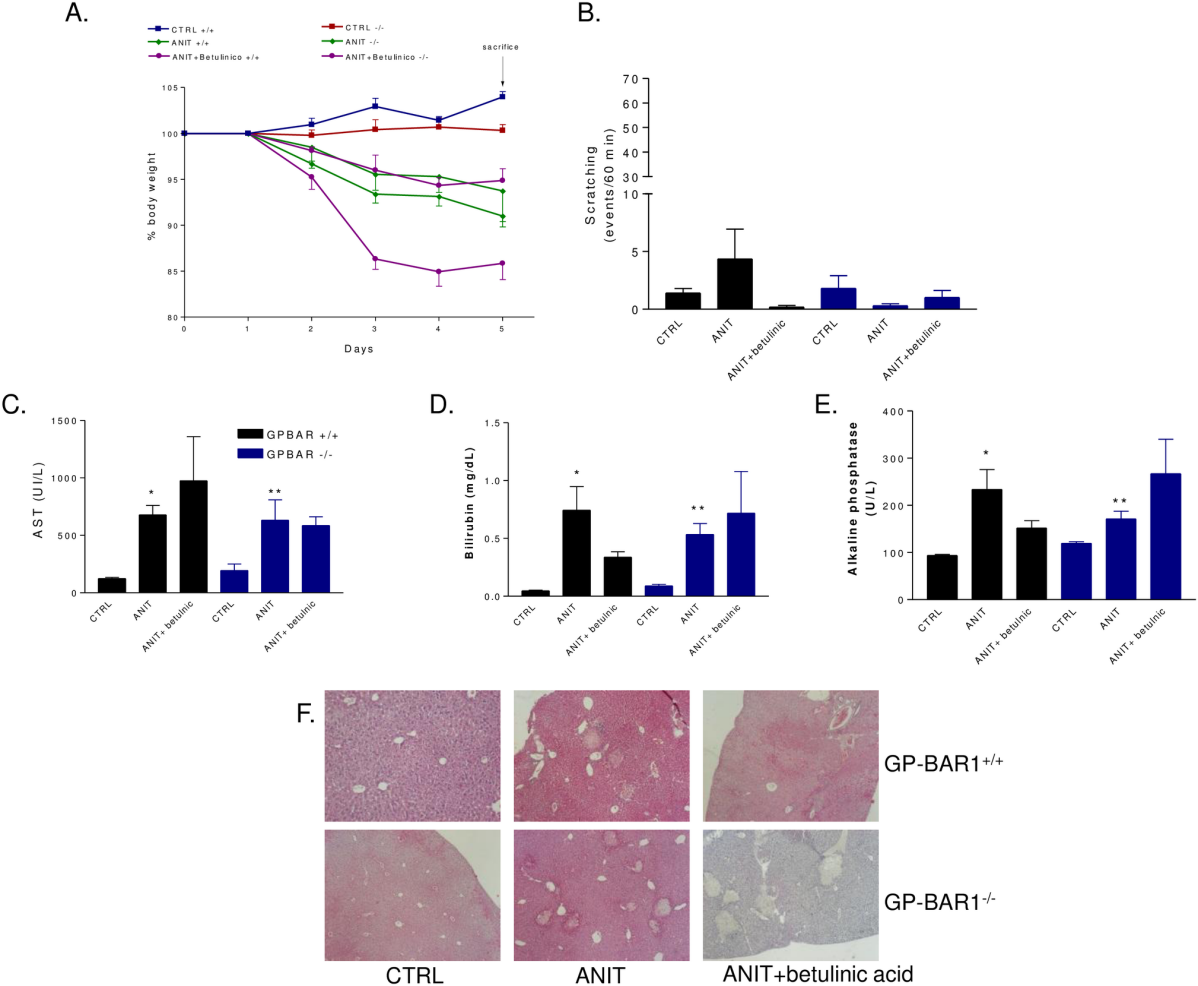
Effect of betulinic acid on ANIT induced cholestasis in GPBAR1^+/+^ and-^/-^ mice. GPBAR1^**+/+**^ and GPBAR1^**-/-**^ mice were administered with ANIT or with the combination of ANIT plus betulinic acid for 5 days. (A) Body weight; (B) Scratching test was performed at day 5. Results are expressed as the number of scratching events during 60 minutes of observation. Results are the mean ± SE of 4–8 mice per group. (C) Serum levels of AST, (D) Bilirubin and (E) Alkaline phosphatase. Results are the mean ± SE of 4–8 mice per group. *p<0.05 versus GP-BAR^**+/+**^ mice. **p<0.05 versus GPBAR1^**-/-**^ mice. (F) Representative histological pictures of H&E-stained livers.

To further explore the role of GPBAR1 in cholestasis, we have then investigated whether betulinc acid triggers or exacerbates itching when administered systemically to mice rendered cholestatic by ANIT. As shown in Fig 6, betulinic acid failed to protect against liver damage caused by ANIT in wild type mice as assessed by measuring AST plasma levels and histopathology scores, but did not induced spontaneous itching (Fig 6B).

### BAR502 protects against estrogen induced cholestasis in mice

**Fig 7:**
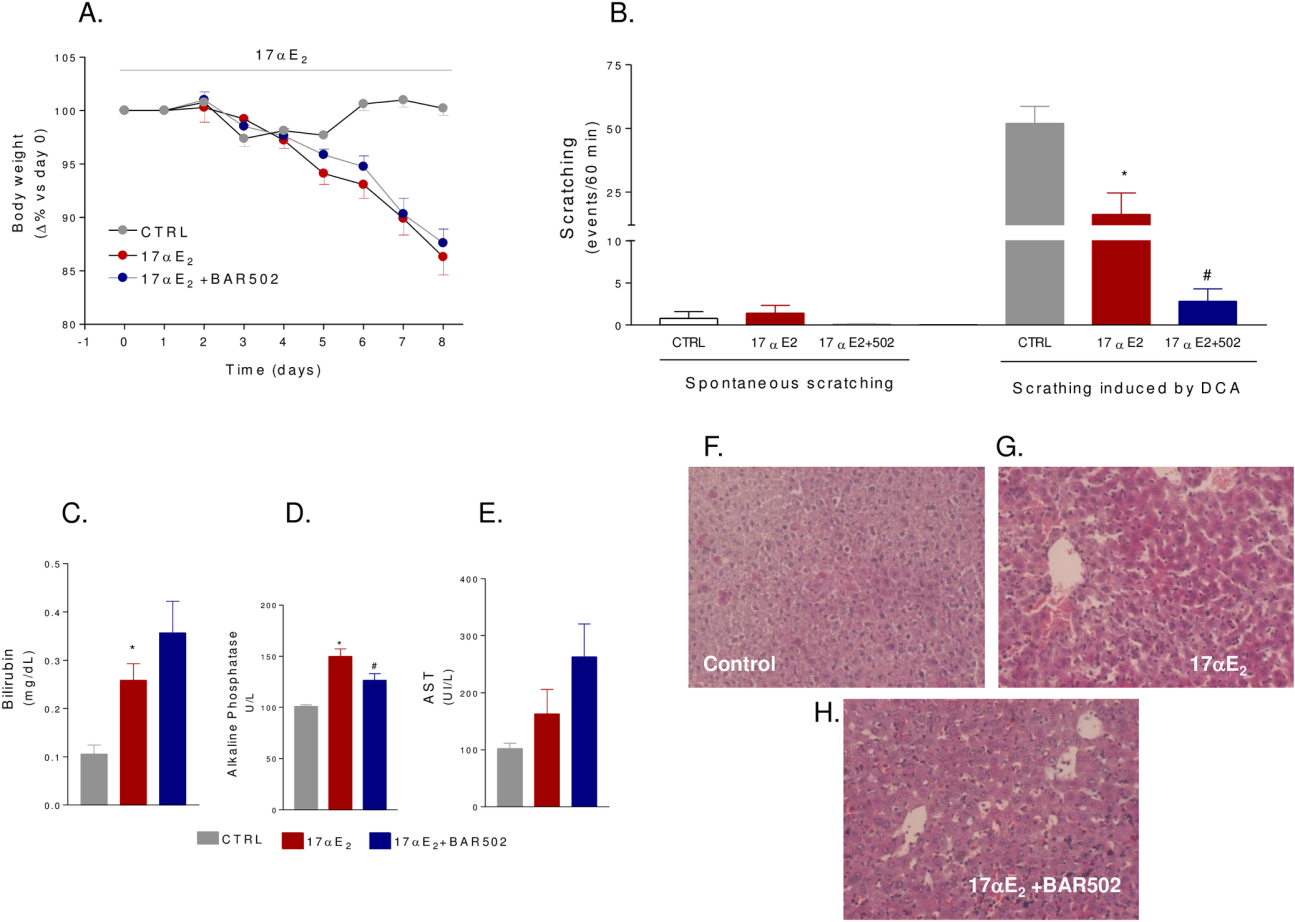
Effect of BAR502 on cholestasis induced by 17αE_2_. C57BL6 mice were treated for 8 days with 17αE_**2**_ or with the combination of 17αE_**2**_ plus BAR502 as described in materials and methods. (A) Body weight. (B) Scratching tests were performed at day 8. Mice were subjected to intradermal injection of DCA and scratching events were measured during 60 minutes of observation. (C) Serum levels of bilirubin. (D) Serum levels of Alkaline phosphatase. (E) Serum levels of AST. Results are the mean ± SE of 4–8 mice per group. *p<0.05 versus control group (CTRL). #p<0.05 versus 17αE_**2**_ treated mice. (F-H) Representative histological pictures of H&E-stained livers.

**Fig 8:**
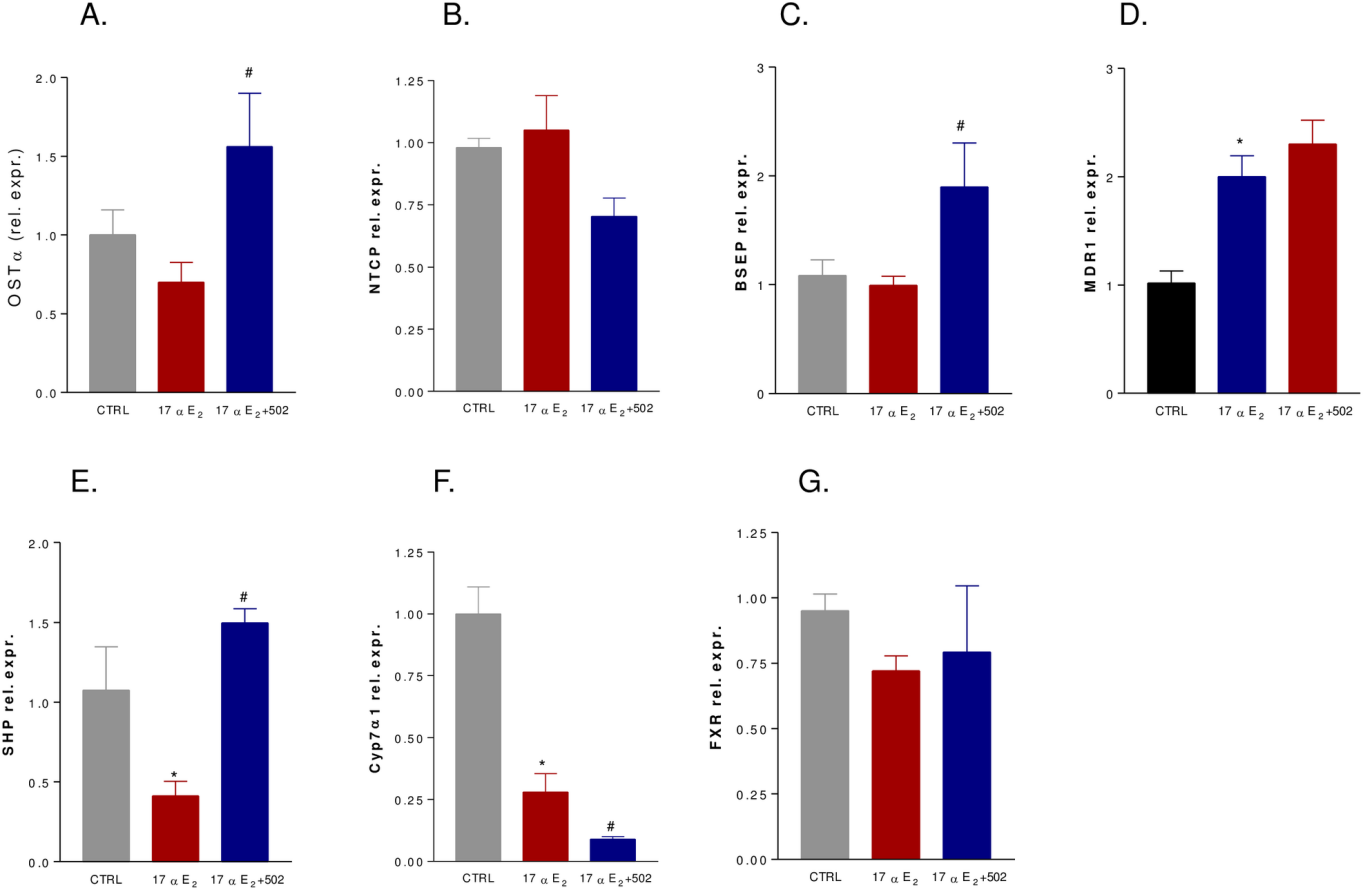
Effects of administration of BAR502 on cholestasis induced by 17αE_2_ for 8 days on relative mRNA expression of (A) OSTα, (B) NTCP, (C) BSEP, (D) MDR1, (E) SHP, (F) Cyp7α1 and (G) FXR. Values are normalized relative to GAPDH mRNA and are expressed relative to those of control animals, which were arbitrarily set to 1. Results are the mean ± SE of 4–8 mice per group. *p<0.05 versus control group (CTRL); #p<0.05 versus 17αE_**2**_ treated mice.

Cholestasis induced by administration of 17αE_2_, a bio-active synthetic estrogen, is a model for ICP[32]. In this set of experiments, C57BL6 GPBAR1^+/+^ mice (congenic to GPBAR1^-/-^ mice) were administered 17αE_2_, 10 mg/kg/day, alone or in combination with BAR502, 15 mg/kg/day, for 8 days. As illustrated in Fig 7A, 17αE_2_ caused a body weight reduction and this pattern was not reversed by BAR502 (Fig 7A, p<0.05 for both groups versus baseline). A scratching test was performed in these animals on day 8. As shown in Fig 7B, spontaneous scratching did not increased in response to treatment with 17αE_2_ or the combination of 17αE_2_ plus BAR502. Subcutaneous injection of DCA strongly stimulated scratching within 60 minutes in naαve mice, but this response was attenuated in mice administered 17αE_2_ alone or in combination with BAR502 (Fig 7B, p<0.05). 17αE_2_ treatment led to a significant increase of serum levels of bilirubin and alkaline phosphatase (Fig 7C and 7D, p<0.05) but not of AST (Fig 6E). These changes were attenuated by co-treating mice with BAR502 (Fig 7D, p<0.05). At the histopathology analysis, livers from mice treated with 17αE_2_ had elongated bile ducts with dilated lumina and irregular epithelium surrounded by edematous portal connective tissue infiltrated by neutrophils (Fig 7F and 7G). BAR502 attenuated bile duct enlargement and neutrophil infiltration (Fig 7H). As shown in Fig 8, 17αE_2_ administration did not alter the relative mRNA expression of basolateral transporters OSTα and NTCP as well as that of the canalicular transporter BSEP (Fig 8A–8C). In contrast, BAR502 increased the expression of OSTα and BSEP (Fig 8A and 8C, p<0.05). Finally, the investigation of SHP and Cyp7α1 mRNA levels confirmed that during cholestasis the expression of both genes is downregulated and that BAR502 effectively induces SHP mRNA expression while repress Cyp7α1 (Fig 8E and 8F, p<0.05).

### Effect of BAR502 on bile acid pool in the model of estrogen-induced cholestasis

**Fig 9:**
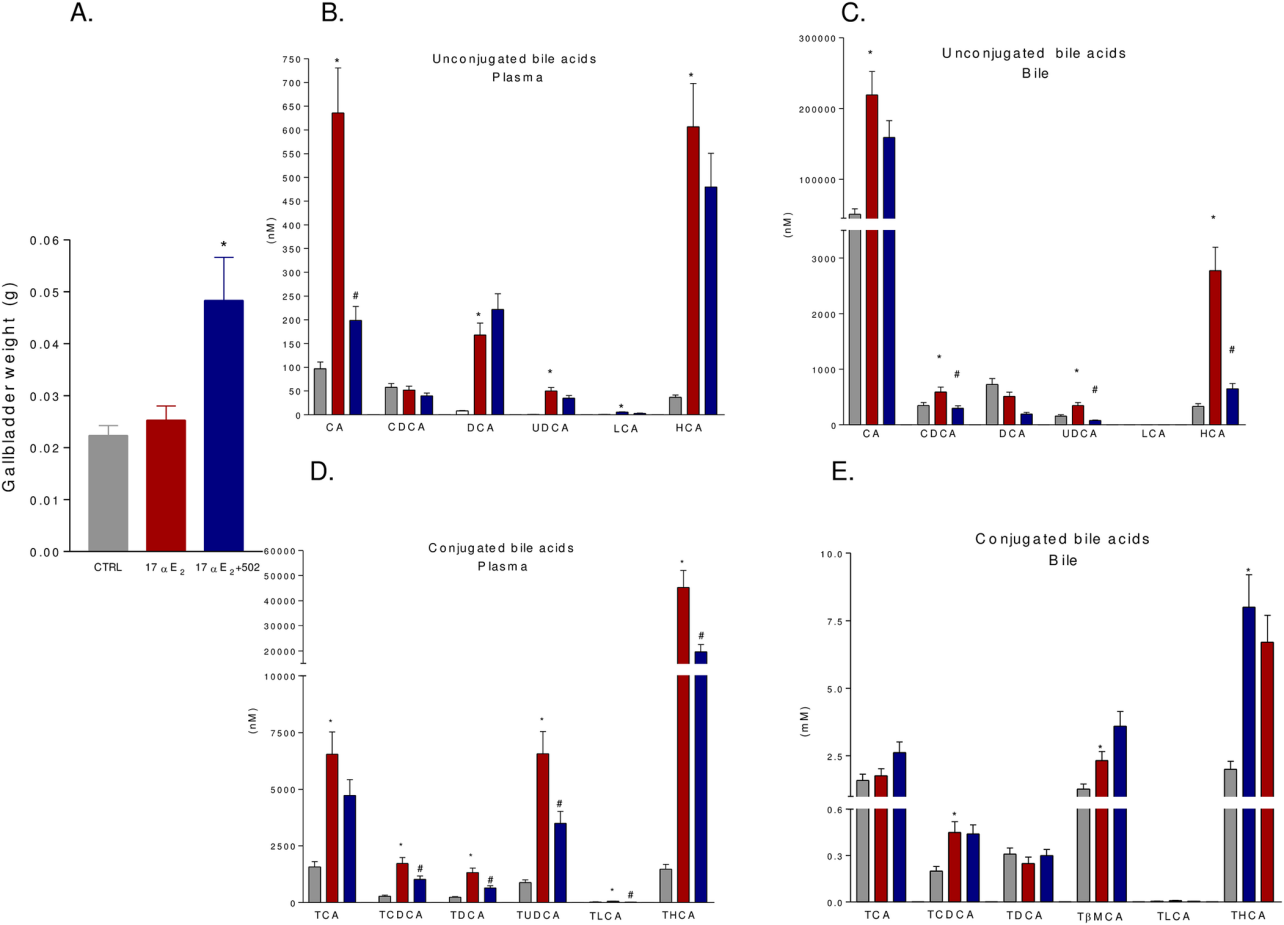
Quantitative evaluation of serum and gallbladder composition of bile acids after administration of 17αE_2_ alone or 17αE_2_ plus BAR502. (A) Gallbladder weight. (B-C) Quantitative evaluation of serum (B) and gallbladder (C) non conjugated bile acids from control animals (CTRL), animals treated with 17αE_**2**_ or animals co-administered with 17αE_**2**_ plus BAR502. (D-E) Quantitative evaluation of serum (D) and gallbladder (E) conjugated bile acids from either control animals (CTRL), animals treated with 17αE_**2**_ or animals co-administered with 17αE_**2**_ plus BAR502. Results are the mean ± SE of 4–8 animals per group. *p<0.05 versus control group (CTRL); #p<0.05 versus 17αE_**2**_ treated mice.

Administration of BAR502 resulted in two-fold increase of gallbladder weight, a GPBAR1 dependent effect (Fig 9A, p<0.05). In addition, BAR502 administration had a robust impact on bile acid pool. Thus, plasma concentrations of non conjugated bile acids, CA, DCA, UDCA and HCA were significantly increased by 17αE_2_ treatment (Fig 9B, p<0.05). Co-administration of BAR502 reduced CA while levels of DCA, UDCA and HCA were substantially stable (Fig 8B, p<0.05). Similarly to what observed in the serum, gallbladder content of non conjugated bile acids, CA, CDCA, UDCA and HCA, was increased by 17αE_2_ and BAR502 coadministration reduced CDCA, UDCA and HCA but not CA (Fig 9C, p<0.05). The analysis of conjugated bile acids in the serum confirmed these data. As shown in Fig 9D, 17αE_2_ increased serum concentrations of TCA, TCDCA, TDCA, TUDCA, TLCA and THCA (p<0.05) and BAR502 was effective in reducing TCDCA, TDCA, TUDCA, TLCA, THCA but not TCA (Fig 9D, p<0.05). Analysis of conjugated bile acids in the gallbladder revealed that 17αE_2_ administration increases the levels of TCDCA, TLCA, TβMCA and THCA but not that of TCA and TDCA (Fig 9E, p<0.05). Noteworthy, co-administration of BAR502 reduced gallbladder content of TLCA (Fig 9E, p<0.05). Relative percentage of bile acids (non conjugated and conjugated) in the plasma and gallbladder is shown in S2 and S3 Figs.

## Discussion

In the present study we report the pharmacological characterization of BAR502, a non bile acid steroidal ligand for GPBAR1 and FXR, i.e two bile acid activated receptors. In vitro studies shown in Fig 1 demonstrate that BAR502 effectively transactivates FXRE in HepG2 cells, and CRE in HEK293T cells, with an EC_50_ of 2 μM and 0.4 μM, respectively. Consistent with its ability to transactivate both FXR and GPBAR1, BAR502 increased the expression of OSTα, BSEP and SHP, three canonical FXR target genes, in HepG2 cells and triggered the accumulation of cAMP in THP-1 cells and the release of GLP-1 from Glutag cells, a validated test for GPBAR1 agonism. Because, in comparison to conventional bile acids the COOH group in position C23 is substituted by an OH group, BAR502 represents the prototype of a novel class of non bile acid, steroidal, dual ligands for GPBAR1 and FXR.

Since dual FXR and GPBAR1 ligands represent a promising opportunity in the treatment of liver disorders, we have then investigated whether BAR502 attenuates cholestasis and itching in rodent models of cholestasis. Itching is the main symptom of cholestasis thought its pathogenesis remains elusive[33]. Thus, despite dermal injection of bile acids triggers itching [34–36], the severity of the symptom shows poor correlation with plasma bile acids concentrations and other mediators, such lysophospatidic acid, demonstrate a better correlation with itching severity [37].

Here we report that intradermal injection of DCA, LCA and betulinic acid in mice induces itching in a GPBAR1 dependent manner, but this signaling pathway is desensitized by re-challenging mice with the same agent. Despite we have not investigated the molecular mechanisms that support this finding, there is evidence that pruritus induced by bile acids requires, in addition to GPBAR1, the co-activation of multiple receptors and mediators including TRPA1 channels [38]. GPBAR1 and TRPA1 channels are co-expressed in cutaneous afferent neurons and their activation trigger the release of CGRP, a pruritogenic peptide.^38^ Thus, while GPBAR1 signaling *per se* is thought to be resistant to desensitization[39], TRPA1 channels and CGRP receptor undergo desensitization in response to repeated stimulation [40].

One important observation made in the present study is that, in contrast to intact animals, in mice administered ANIT or 17αE_2_ intradermal administration of DCA fails to elicit a itching behavior. The fact that cholestatic mice become unable to respond to itching has multiple explanations. In addition to alteration of GPBAR1-mediated activation of TRPA1-CGRP pathways, GPBAR1 agonism activates also opioid mechanisms in the spinal cord causing analgesia toward somatic pain, a mechanism that might result in impaired perception of DCA-induced itching in the setting of chronic cholestasis, i.e. when concentrations of endogenous bile acids-including GPBAR1 ligands- are chronic elevated [27]. In addition to this opiod-related mechanism, it well known that severe liver disorders cause analgesia, and a painless jaundice is observed commonly in end-stage liver disorders, due to multiple defects including alterations in the state of consciousness, i.e. hepatic encephalopathy, that typically occurs in this clinical setting [41,42]. Despite the fact that, for ethical reasons, somatic pain was not investigated in our mice models of cholestasis, decrease in animals well being could support the impaired ability of ANIT- and 17αE_2_-treated mice to elicit a itching behavior when challenged with DCA.

Confirming this view we found that betulinic acid, a GPBAR1 ligand, failed to trigger pruritus in cholestatic mice, thus suggesting that activation of GPBAR1 *per se* might not be the cause of itching observed in patients suffering for chronic cholestasis.

The liver injury caused by ANIT is a model for drug-induced cholestasis and is thought to reflect the potential toxicity of this chemical for hepatocytes and biliary cells [31]. In the present study we have shown that liver injury caused by ANIT was exacerbated by GPBAR1 gene ablation. At the dose of 25 mg/kg, ANIT caused 100% mortality over a period of 10 days in GPBAR1^-/-^ mice compared with 40% lethality in GPBAR1^+/+^ mice. Co-treating mice with BAR502 rescued GPBAR1^-/-^ mice from death caused by ANIT and attenuated the extent of liver injury as measured by AST plasma levels and histopathology scores. These beneficial effects were FXR mediated as demonstrated by the fact that BAR502, robustly reshaped the liver expression of FXR target genes. ANIT administration reduced the liver expression of SHP and increased the expression of MDR1, while the expression of FXR, BSEP, NTCP, OSTα and Cyp7A1 was substantially unchanged in both wild type and GPBAR1^-/-^ mice, administering mice with BAR502, resulted in a robust induction of SHP, OSTα and BSEP in both wild type and GPBAR1^-/-^ mice, while the expression of MDR1 and Cyp7A1 was reduced. Because these changes are compatible with FXR activation [20], it appears that BAR502 behaves as an effective FXR ligand *in vivo* in both wild type and GPBAR1^-/-^ mice.

The cholestasis induced by 17αE_2_ is a model for ICP.^32^ Administration of BAR502 in this model reduced biochemical makers of cholestasis (alkaline phospatase) and induced a pattern of expression of FXR target genes that is consistent with the activation of this nuclear receptor in the liver [24,29]. Similarly to the ANIT model, animals rendered cholestatic by exposure to 17αE_2_ failed to show spontaneous bouts of scratching and the scratching response caused by intradermal injection of DCA was robustly attenuated in comparison to naïve mice. Treatment with BAR502 did not caused spontaneous scratching and reversed the scratching behavior caused by intradermal injection of DCA. Importantly treating mice with BAR502 resulted in a profound modification of bile acid pool with a marked reduction of plasma and bile concentrations of CA, TCA, HCA, TCDCA, TDCA and THCA. Interesting, administration of BAR502 increased the gallbladder weight, a pharmacological effect that has been linked to GPBAR1 activation [43].

In summary, we have shown that while acute injection of GPBAR1 ligands in the skin of naïve mice trigger itching bouts, this pathway seems to be completely deactivated in cholestasis. Selective GPBAR1 ligands and dual FXR/GPBAR1 ligands do not cause itching in rodent model of cholestasis. Consistent with these findings BAR502, a dual FXR/GPBAR1 ligand, attenuates liver damage in animal models of non-obstructive cholestasis without inducing itching.

## Supporting Information

S1 Fig Effects of 6-ECDCA on scratching in GPBAR1^+/+^ and GPBAR1^-/-^ mice. GPBAR1^+/+^ and GPBAR1^-/-^ mice were subjected to intradermal injection of 6-ECDCA at the dose of 25 μg. Results are expressed as the number of scratching events during 60 minutes of observation. Results are the mean ± SE of 4 mice per group. *p<0.05 versus control group; **p<0.05 versus GPBAR1^+/+^ mice.
(TIF)

S2 Fig Effect of BAR502 on qualitative analysis of plasma conjugated and non conjugated bile acids from control mice (CTRL), 17αE_2_ administered mice (17αE_2_) and mice coadministered with 17αE_2_ plus BAR502. (TIF)

S3 Fig Effect of BAR502 on qualitative analysis of gallbladder content of conjugated and non conjugated bile acids from control mice (CTRL), 17αE_2_ administered mice (17αE_2_) and mice coadministered with 17αE_2_ plus BAR502. (TIF)
