# Supplementary figures and images for: Impaired Itching Perception in Murine Models of Cholestasis Is Supported by Dysregulation of GPBAR1 Signaling
Source: PLoS One. 2015 Jul 15;10(7):e0129866. doi: 10.1371/journal.pone.0129866 (PMC4503431; doi:10.1371/journal.pone.0129866)

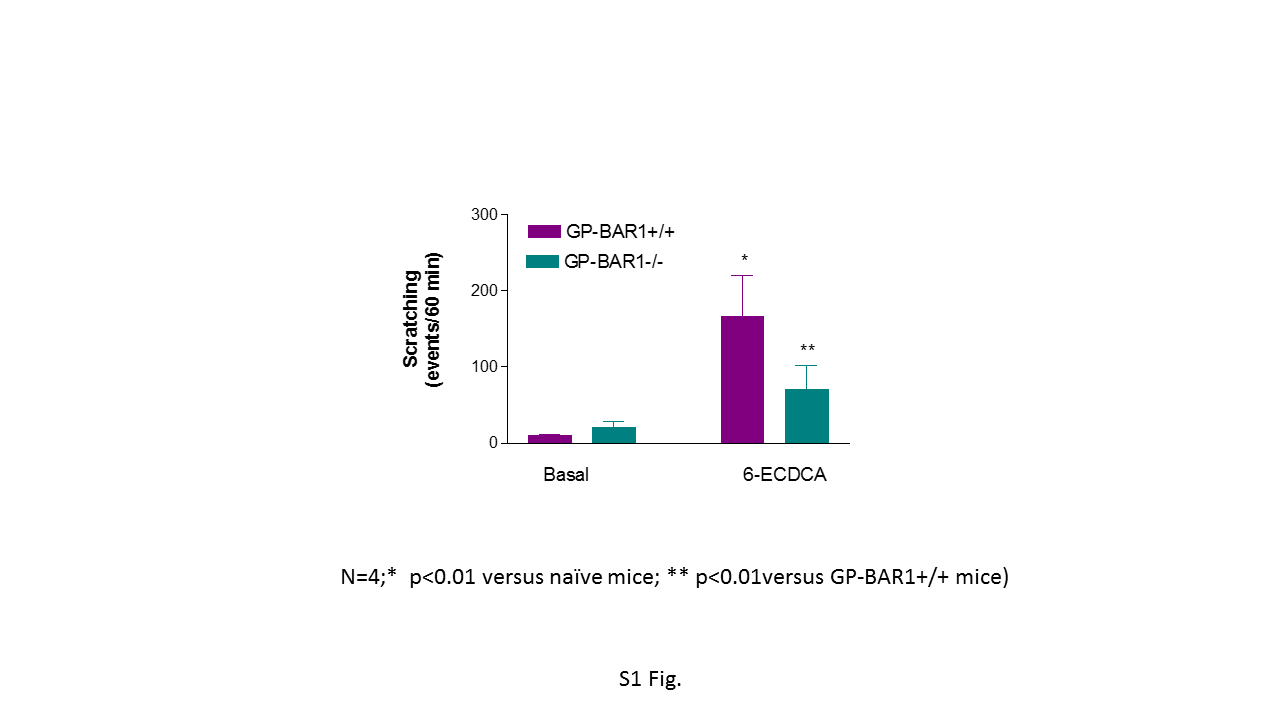

Supplement: S1 Fig — GPBAR1+/+ and GPBAR1-/- mice were subjected to intradermal injection of 6-ECDCA at the dose of 25 μg. Results are expressed as the number of scratching events during 60 minutes of observation. Results are the mean ± SE of 4 mice per group. *p<0.05 versus control group; **p<0.05 versus GPBAR1+/+ mice. (TIF) [file pone.0129866.s001.tif]

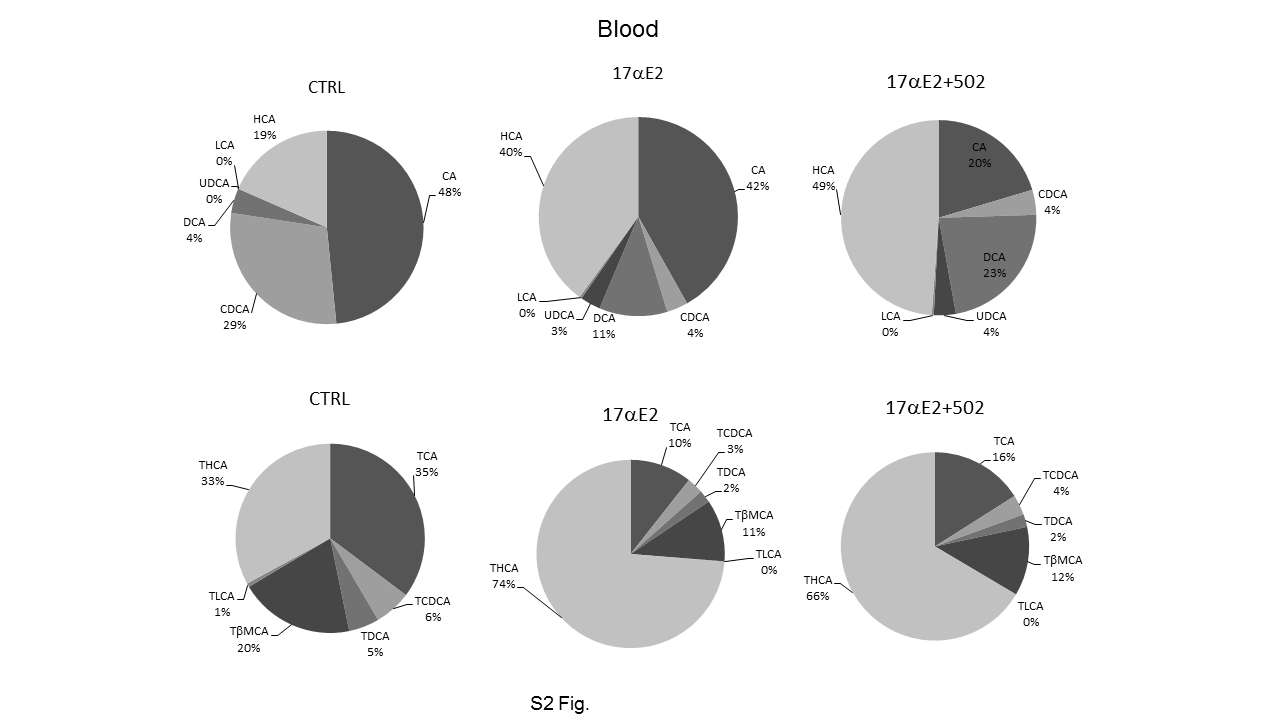

Supplement: S2 Fig — (TIF) [file pone.0129866.s002.tif]

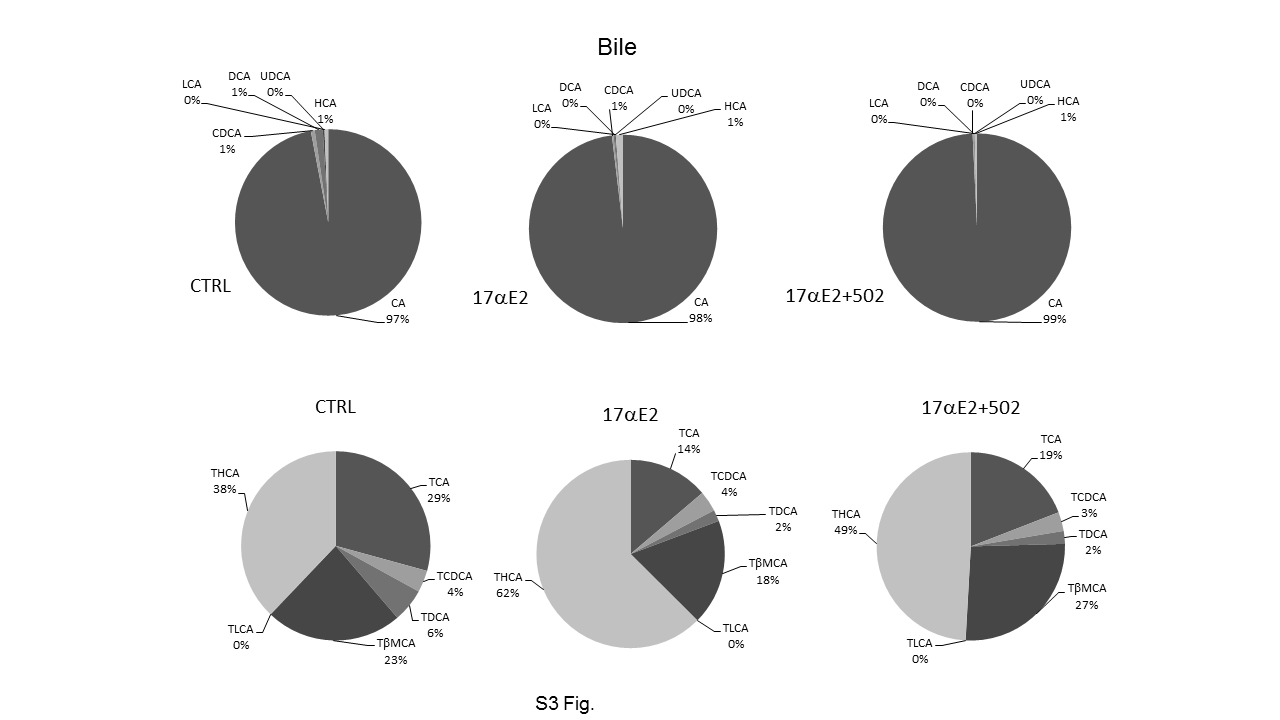

Supplement: S3 Fig — (TIF) [file pone.0129866.s003.tif]
